# Structural Properties Dictating Selective Optotracer Detection of *Staphylococcus aureus*

**DOI:** 10.1002/cbic.202100684

**Published:** 2022-04-01

**Authors:** Karen Butina, Linda Lantz, Ferdinand X. Choong, Ana Tomac, Hamid Shirani, Susanne Löffler, K. Peter R. Nilsson, Agneta Richter‐Dahlfors

**Affiliations:** ^1^ AIMES – Center for the Advancement of Integrated Medical and Engineering Sciences Karolinska Institutet and KTH Royal Institute of Technology Solnavägen 9 171 77 Stockholm Sweden; ^2^ Department of Neuroscience Karolinska Institutet Solnavägen 9 171 77 Stockholm Sweden; ^3^ Department of Chemistry IFM Linköping University 581 83 Linköping Sweden

**Keywords:** bacterial detection, fluorescence microscopy, fluorescence spectroscopy, optotracers, real-time monitoring

## Abstract

Optotracers are conformation‐sensitive fluorescent tracer molecules that detect peptide‐ and carbohydrate‐based biopolymers. Their binding to bacterial cell walls allows selective detection and visualisation of *Staphylococcus aureus* (*S. aureus*). Here, we investigated the structural properties providing optimal detection of *S. aureus*. We quantified spectral shifts and fluorescence intensity in mixes of bacteria and optotracers, using automatic peak analysis, cross‐correlation, and area‐under‐curve analysis. We found that the length of the conjugated backbone and the number of charged groups, but not their distribution, are important factors for selective detection of *S. aureus*. The photophysical properties of optotracers were greatly improved by incorporating a donor‐acceptor‐donor (D‐A‐D)‐type motif in the conjugated backbone. With significantly reduced background and binding‐induced on‐switch of fluorescence, these optotracers enabled real‐time recordings of *S*. *aureus* growth. Collectively, this demonstrates that chemical structure and photophysics are key tunable characteristics in the development of optotracers for selective detection of bacterial species.

## Graphical abstract

**Optotracers** are conformation‐sensitive fluorescent tracer molecules that detect peptide‐ and carbohydrate‐based biopolymers. In this study, we investigated the structural properties providing optimal detection of *Staphylococcus aureus*. We show that the chemical structure and photophysics of these molecules are key tunable characteristics in the development of optotracers for selective detection of bacterial species.

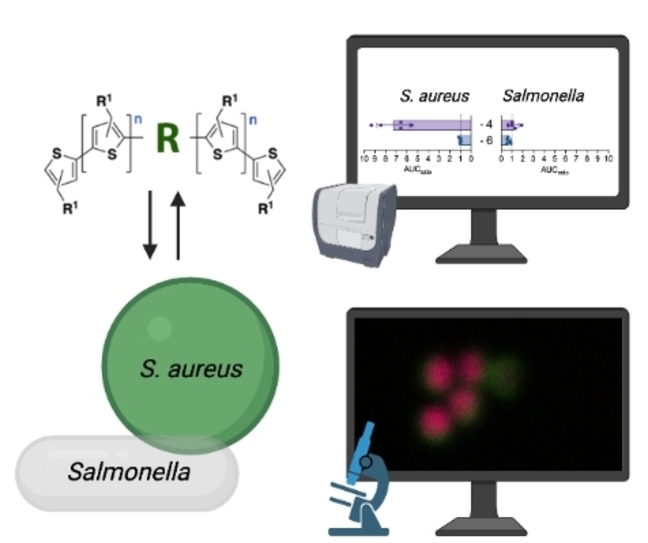

## Introduction

Optotracers have recently gained attention as fluorescent tracer molecules in the field of microbiology. By enabling spectral assignment of bacterial amyloid fibrils and cellulose, optotracers were shown as real‐time reporters of biofilm formation by *Salmonella enterica* serovars Enteritidis (*S*. Enteritidis) and Typhimurium.^[1]^ Based on the ability to detect cellulose, optotracing was further developed as a pioneering technology for biofilm diagnostics in urinary tract infections.^[2]^ The molecules used in these studies are derived from luminescent conjugated poly‐ and oligomers (LCPs, LCOs), a class of organic molecules with unique electronic, electrochemical and optical properties originating from the conjugated structure of the compounds, resulting in thermo‐, solvato‐, piezo‐, iono‐ and photochromisms of the material.^[3]^

LCPs and LCOs were originally found to interact with biopolymers such as DNA[^4^, ^5^] and proteins.^[6]^ Binding to biopolymers influences the conformation of the conjugated backbone as well as the molecules’ interchain‐ and side chain interactions, all associated with changes in the optical spectra of the molecules.^[3]^ The defined spectral signatures, derived from binding‐induced alteration of the optical properties,^[7]^ have been extensively used as a proxy for LCO binding to biomolecules such as disease associated protein aggregates in humans, allowing their spectral discrimination.[^8^, ^9^, ^10^] When found to differentiate between components of the extracellular matrix of bacterial biofilms[^1^, ^2^] and polysaccharides in the glycobiology field,[^11^, ^12^, ^13^] the molecules were further investigated as optotracers. Recently, we demonstrated optotracer binding to cell wall components of Gram‐positive bacteria, e. g. peptidoglycan (PGN), thereby enabling selective detection of the pathogen *Staphylococcus aureus* (*S. aureus*).^[14]^ By introducing a donor‐acceptor‐donor (D‐A‐D)‐type electronic structure in the conjugated backbone of the optotracer, an on‐switch of fluorescence occurred, which enabled real‐time detection of bacterial cells in growing cultures.^[14]^

**Figure 1:**
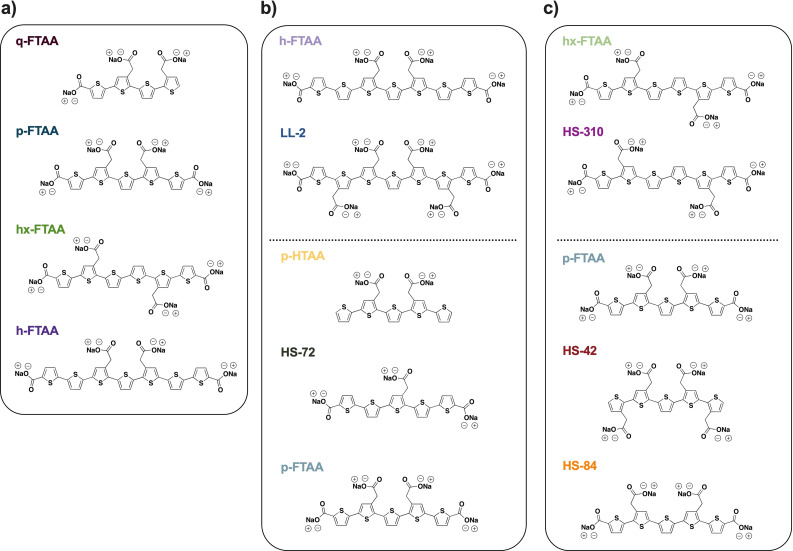
Chemical structures of optotracers. Optotracers with different: a) length of the conjugated backbone (*i. e*. number of thiophene rings), b) total charge, and c) charge distribution along the conjugated backbone. The corresponding sodium salt of each optotracer is shown. The color‐coded names of optotracers correspond to the colour codes used in Figures 2–6.

Here, we investigate the nature of the chemistry that promotes specific binding of optotracers to *S. aureus*. To identify the optimal structural determinants for selective detection of *S. aureus*, we use a large selection of closely related optotracers with different structural properties, such as varying number of thiophene rings (Figure 1a), different total charge of the molecule (Figure 1b), and different charge distribution along the conjugated backbone (Figure 1c). By introducing a D‐A‐D‐type electronic structure, we improved the photophysical characteristics of the molecule such that real‐time measurements during growth of bacterial cultures were achieved. The versatile optotracer‐based detection of *S. aureus* by spectroscopy and microscopy is the first example illustrating how chemical design can be applied to finetune optotracer detection specific for a given bacterial species.

## Results and Discussion

### Effect of the length of the conjugated backbone for bacterial detection

To analyse how the length of the conjugated backbone, defined by the number of thiophene rings, affected bacterial detection, we used the tetra‐, penta‐, hexa‐ and heptameric optotracers q‐FTAA, p‐FTAA, hx‐FTAA and h‐FTAA (Figure 1a). These oligomers were used since previous work on thiophene‐based ligands targeting protein aggregates have identified oligothiophenes comprising 5 to 7 thiophene rings as superior ligands for detection of protein aggregates.^[9]^ First, we characterised the photophysical properties of q‐FTAA (4 thiophene rings) in its free form in PBS, and when incubated with *S. aureus*. The excitation and emission spectra were recorded in a plate reader, and data was visualized in a spectral plot (spec‐plot). To identify the maximum excitation and emission wavelengths (Ex. λ_max_, Em. λ_max_) we used Python programming language to enable automated peak analysis.

**Figure 2:**
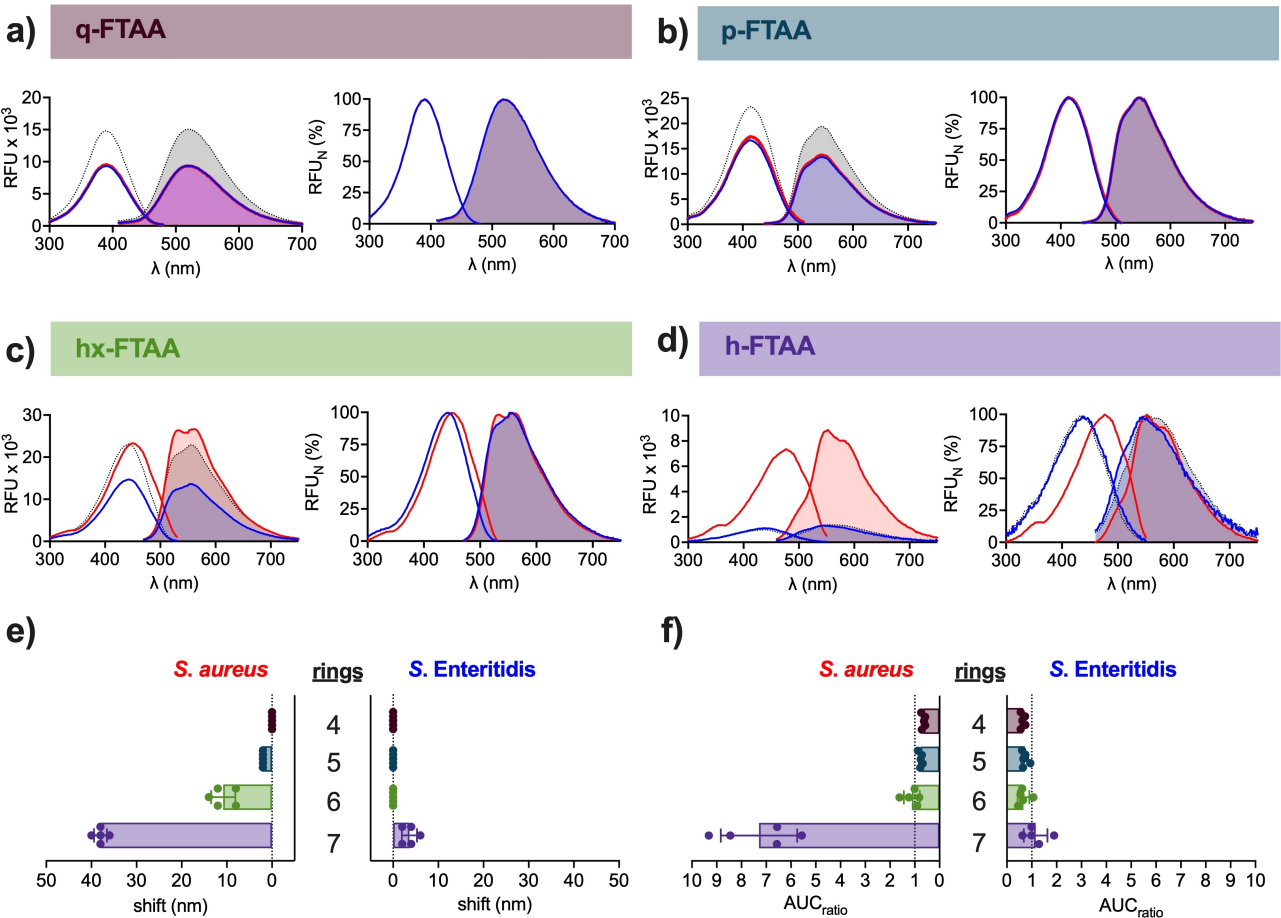
Effect of the length of the conjugated backbone for bacterial detection. Spec‐plots (left) and normalized spec‐plots (right) of: a) q‐FTAA, b) p‐FTAA, c) hx‐FTAA and d) h‐FTAA in free form (dotted line) and when mixed with *S. aureus* (red line) or *S*. Enteritidis (blue line) in PBS. Lines show mean of n=5. e) Spectral shifts calculated by cross‐correlation analysis of the excitation spectra of q‐FTAA (4 rings), p‐FTAA (5 rings), hx‐FTAA (6 rings) and h‐FTAA (7 rings) mixed with *S. aureus* or *S*. Enteritidis. f) The change in fluorescence intensity, calculated as the ratio of the area under curve (AUC_ratio_) of the excitation spectra of q‐FTAA (4 rings), p‐FTAA (5 rings), hx‐FTAA (6 rings) and h‐FTAA (7 rings) mixed with *S. aureus* or *S*. Enteritidis, and the AUC of free optotracer. Bars in (e, f) show mean±SD of n=5. Each individual data point is shown as a filled circle. The color‐coded names of optotracers correspond to the colour codes of bars. Optrotracers were used at 1 μM.

While peak analysis is useful for spectral comparisons, noise may become an issue in low‐intensity spectra. To facilitate comparisons of peak excitation and emission wavelengths between spectra, we prepared a normalized spec‐plot, in which we assigned 0 % to the lowest and 100 % to the highest relative fluorescence unit (RFU) in each sample. Unbound q‐FTAA showed Ex. λ_max_ of 390 nm and Em. λ_max_ of 520.4 nm (Figure 2a, Table S1). When incubating q‐FTAA with *S. aureus*, we found Ex. λ_max_ and Em. λ_max_ to overlap with the free form. As no alterations in the optical spectrum were detected, we conclude that q‐FTAA most likely does not bind this bacterium. To analyse if q‐FTAA detects Gram‐negative bacteria, spectra of this optotracer mixed with *S*. Enteritidis were analysed. Peak values again overlapped with those from free q‐FTAA, suggesting an inability of this optotracer to detect any of the two bacterial species.

As we repeated the experiments using p‐FTAA (5 thiophene rings), spec‐plot analysis identified Ex. λ_max_ 414.4 nm and Em. λ_max_ 543.6 nm for the free form (Figure 2b, Table S1). The spectra of p‐FTAA incubated with *S. aureus* (Ex. λ_max_ 416 nm and Em. λ_max_ 542.8 nm) and *S*. Enteritidis (Ex. λ_max_ 414 nm, Em. λ_max_ 544 nm) nearly overlapped with that of the free optotracer, suggesting that the pentameric optotracer cannot be used for detection of any of the tested bacterial species. A different picture emerged when we analysed hx‐FTAA (6 thiophene rings). Compared to spectra from its free form in PBS (Ex. λ_max_ 442 nm, Em. λ_max_ 556 nm), hx‐FTAA incubated with *S. aureus* showed a red shift of circa 8 nm in Ex. λ_max_ (450.4 nm), and two peaks in the emission spectrum: one at a wavelength similar to that of free hx‐FTAA (Em. λ_max1_ 560.4) and an additional peak at a shorter wavelength (Em. λ_max2_ 532.4 nm) (Figure 2c, Table S1). The red‐shift of Ex. λ_max_ and the appearance of an additional peak in the emission spectrum suggest that the conformation of hx‐FTAA changes in the presence of *S. aureus*, most likely due to binding of the hexameric optotracers to the Gram‐positive cells. In contrast, spectra from hx‐FTAA mixed with *S*. Enteritidis (Ex. λ_max_ 443.2 nm, Em. λ_max_ 556 nm) were nearly identical to that of free hx‐ FTAA, indicating absence of binding. Collectively, this shows that hx‐FTAA is well suited for detection of *S. aureus* but not *S*. Enteritidis.

Analysis of h‐FTAA (7 thiophene rings) showed Ex. λ_max_ at 434.8 nm and Em. λ_max_ at 553.6 nm of the free form (Figure 2d, Table S1). When incubated with *S. aureus*, a large redshift (43 nm) of Ex. λ_max_ (478 nm) was observed, Em. λ_max_ (550.4 nm) remained almost unchanged, and the fluorescence intensity was pronouncedly increased. These spectral changes originate from conformational changes induced upon binding to *S*. *aureus*. In the presence of *S*. Enteritidis, Ex. λ_max_ (435.2 nm) remained unchanged, Em. λ_max_ (545.6 nm) showed a blue shift and the fluorescence intensity remained low, indicating lack of binding to this bacterial species. Thus, incubation of hx‐ and h‐FTAA with *S. aureus* generated changes in the optical spectra indicative of binding to this bacterium. Since such changes were not observed for q‐ and p‐FTAA, it shows that the conjugated backbone must consist of at least 6 rings in order to enable detection of *S. aureus*. Interestingly, the increased length of the conjugated backbone did not confer any ability to detect *S*. Enteritidis, thus demonstrating that detection was selective for *S. aureus*.

### Cross‐correlation analysis for quantification of spectral shifts

Since automated peak analysis only considers Ex. λ_max_ and Em. λ_max_, the majority of information present in the spectrum is not utilized. We hypothesized that use of the entire spectra may help increasing the robustness of spectral shift evaluations, and we therefore employed cross‐correlation analysis. In cross‐correlation, which is commonly used in signal processing, the entire spectrum is shifted to achieve best overlap with the control, thereby providing an objective and quantitative value for the spectral shift. This allows easy interpretation of results with no need of further analysis. Importantly, cross‐correlation analysis almost eliminates the influence of noise. When analysing spectra in Figure 2a–d by cross‐correlation, we focused on the excitation spectra since the automated peak analysis had shown differences predominantly in Ex. λ_max_. Cross‐correlation of spectra from free optotracer and from optotracers mixed with *S. aureus* showed no shift for q‐FTAA, while spectral shifts were observed for p‐FTAA (2±0 nm), hx‐FTAA (10.8±2.7 nm) and h‐FTAA (38±1.4 nm) (Figure 2e, Table S2). These results correlated well with spectral shifts obtained by automated peak analysis (Figure 2a–d, Table S1). When mixed with *S*. Enteritidis, cross‐correlation of spectra from tetra‐, penta‐, and hexameric optotracers showed no shifts compared to the free form, whereas a small shift (3.6±1.7 nm) was observed for the heptameric optotracer by cross‐correlation but not automated peak analysis. This discrepant result can be explained by a slight misalignment of spectra from h‐FTAA in free form and when mixed with *S*. Enteritidis (Figure 2d). The ability to obtain precise information on the peak locations as well as the size of spectral shifts illustrate how automated peak analysis and cross‐correlation analysis complement each other.

### Area under curve (AUC) analysis for quantification of total fluorescence intensity

In addition to red‐shifted Ex. λ_max_, an increase in fluorescence intensity was observed for h‐FTAA incubated with *S. aureus* (Figure 2d). Increased fluorescence, which is commonly observed for optotracers binding amyloids, results from binding‐induced alteration of the immediate microenvironment.^[9]^ To quantify the change in fluorescence intensity, we calculated area under the curve (AUC) of aligned excitation spectra, thereby obtaining a value of total fluorescence intensity for optotracers in free form (AUC_optotracer_) and when mixed with *S. aureus* or *S*. Enteritidis (AUC_sample_), respectively. The fluorescence ratio (AUC_ratio_) was then calculated by AUC_sample_/AUC_optotracer_. When mixed with *S*. *aureus*, AUC_ratio_<1 was observed for q‐FTAA (0.65±0.08) and p‐FTAA (0.76±0.07) (Figure 2f, Table S2). The AUC_ratio_ of hx‐FTAA was approximately the same in the absence and presence of *S. aureus* (1.12±0.32), while a circa 7‐fold increase was observed for h‐FTAA incubated with *S. aureus*. No changes related to AUC_ratio_ were observed for any optotracer mixed with *S*. Enteritidis.

### Correlation of optotracer spectroscopy and microscopy

In spectroscopy, any observed spectral shift originates from fluorescence in the bulk of the sample. In contrast, microscopy reveals the fluorescence from individual bacterial cells. To investigate how the two methods correlate, we added q‐FTAA, p‐FTAA, hx‐FTAA and h‐FTAA to mixed samples of *S. aureus* and *S*. Enteritidis, then imaged the mix under a confocal microscope. Micrographs showed distinct fluorescence of the coccoid *S*. *aureus* when incubated with hx‐FTAA and h‐FTAA, while none of the optotracers detected the rod‐shaped *S*. Enteritidis (Figure S1a). These results corroborate the findings from spectral analysis which indicate selective binding of the optotracers to *S. aureus*. Also, these data show that the spectrophotometrically observed small, spectral shifts of p‐FTAA mixed with *S. aureus* and h‐FTAA mixed with *S*. Enteritidis are insufficient to obtain a visual signal under the microscope. Collectively, this shows that binding is required to cause an accumulation of optotracers on bacteria, allowing cells to be visualized. Also, it explains why visualization of the bacterial species is dependent on the concentration of optotracer molecules.

Taken together, results from the above sections show that an extended thiophene backbone enables optotracer‐based detection of *S. aureus*. Binding to bacterial cells is reflected in the red shifts of Ex. λ_max_. Redshifted excitation and absorption spectra have previously been shown to result from binding induced planarization of optotracers.^[15]^ If optotracers bind their targets as individual molecules, we propose that an increased number of thiophene rings in the conjugated backbone leads to stronger aromatic interactions with PGN and possibly other cell wall components, thereby enabling fluorescence detection of *S. aureus*. Aromatic interactions are known to play a major role in the binding of wheat germ agglutinin to PGN^[16]^ as well as in interactions with sugars, despite the hydrophilic nature of the latter.[^16^, ^17^] Moreover, since the penta‐, hexa‐ and heptameric optotracers tested are equally charged, the hydrophobicity of individual molecules increased with increasing length. This supports our previous observation, showing the importance of hydrophobic interactions for binding to and detection of *S. aureus*.^[14]^

### The charge of the optotracer influences detection of *S. aureus*

**Figure 3:**
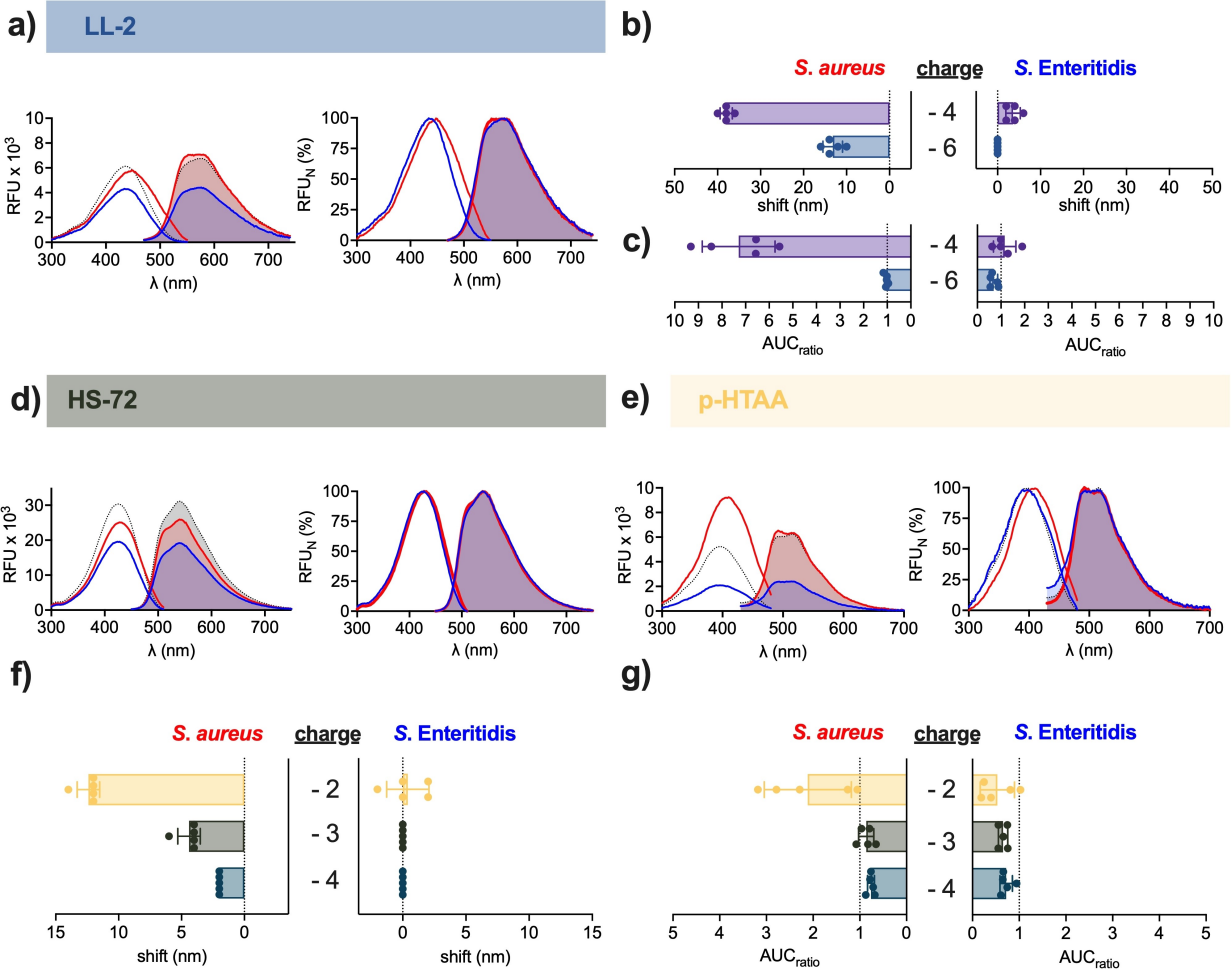
Influence of the total charge for optotracer detection of *S. aureus*. Spec‐plots (left) and normalized spec‐plots (right) of: a) LL‐2, d) HS‐72, and e) p‐HTAA in free form (dotted line) and mixed with *S. aureus* (red line) or *S*. Enteritidis (blue line). Lines show mean of n=5. (b, f) Spectral shifts calculated by cross‐correlation analysis of the excitation spectra of b) LL‐2 (6 negative charges) and h‐FTAA (4 negative charges) and of: f) p‐HTAA (2 negative charges), HS‐72 (3 negative charges), and p‐FTAA (4 negative charges) mixed with *S. aureus* or *S*. Enteritidis. (c, g) The change in fluorescence intensity, calculated as the ratio of the area under curve (AUC) of the excitation spectra of: c) LL‐2 and h‐FTAA, and of g) p‐HTAA, HS‐72, and p‐FTAA, mixed with *S. aureus* or *S*. Enteritidis (AUC_sample_), and the AUC of free optotracer (AUC_optotracer_). Bars in (b, c, f, g) show mean of n=5±SD, filled circles show individual data points. The color‐coded names of optotracers correspond to the colour codes of bars. Optrotracers were used at 1 μM.

We next analysed the influence of the optotracers’ total charge for optimal detection of *S*. *aureus*. Since h‐FTAA had shown strongest binding, recorded as the largest red shift and strongest increase in total fluorescence (see Figure 2e, f), we compared this heptameric molecule with 4 negative charges, to the closely related heptamer LL‐2 whose two additional acetate groups give 6 negative charges (Figure 1b). Automated peak analysis of spectra from the free form of LL‐2 showed Ex. λ_max_ 436.4 nm and Em. λ_max_ 573.6 nm (Figure 3a, Table S1). The Ex. λ_max_ is nearly identical to that of free h‐FTAA, suggesting that additional negative charges have negligible effect on peak excitation of the free molecules. However, a wider Stokes shift was observed for LL‐2 (137 nm) than for h‐FTAA (119 nm), indicating that LL‐2 would be better suited for biological imaging. When incubated with *S*. *aureus*, LL‐2 showed a 10.8 nm redshift of Ex. λ_max_ (447.2 nm) compared to the free form, and a plateaued, dual‐peak emission (Em1. λ_max_ 558.4 nm, Em2. λ_max_ 574.8 nm) (Figure 3a). The shift in Ex. λ_max_ was corroborated by cross‐correlation analysis, showing a 13.2 nm redshift (Figure 3b, Table S2). In contrast, no shifts were observed for LL‐2 mixed with *S*. Enteritidis (Ex. λ_max_ 436.4 nm, Em. λ_max_ 573.6 nm) compared to the free form. When analysing the total fluorescence intensity of LL‐2 mixed with either *S. aureus* or *S*. Enteritidis, relative to that of free optotracer, no changes were observed (Figure 3c, Table S2). This is in stark contrast to h‐FTAA, showing 7‐fold increase when mixed with *S. aureus*. Taken together, the 3‐fold reduction in spectral shift and complete lack of fluorescence increase for LL‐2 compared to h‐FTAA show that increasing the number of negative charges from 4 (h‐FTAA) to 6 (LL‐2) reduces the ability of heptameric optotracers to detect *S. aureus*.

We next analysed if fewer charges would lead to improved detection of *S. aureus*. To this end, we analysed if the minor shift of 2 nm observed from p‐FTAA (4 negative charges) mixed with *S. aureus* (Figure 2b) could be increased by reducing the number of negative charges along the thiophene backbone. Peak analysis of spectrophotometric recordings of the free form of HS‐72 (3 negative charges, Figure 1b) showed Ex. λ_max_ 425.6 nm and Em. λ_max_ 540 nm (Figure 3d). When incubated with *S*. *aureus*, a minor 3.2 nm red shift was observed in Ex. λ_max_ (428.8 nm) compared to the free form, while Em. λ_max_ (540.4 nm) remained the same. Similarly, incubation of HS‐72 with *S*. Enteritidis generated peaks (Ex. λ_max_ 425.2 nm, Em. λ_max_ 540 nm) identical to the free form. This showed that a reduction from 4 to 3 negative charges did not significantly improve bacterial detection. Next, we tested p‐HTAA that has 2 negative charges (Figure 1b). Peak analysis showed Ex. λ_max_ 395.6 nm and a plateaued, dual‐peak emission (Em1. λ_max_ 494.4 nm and Em2. λ_max_ 514.8 nm) in its free form (Figure 3e). When incubated with *S*. *aureus*, Ex. λ_max_ appeared at 408 nm, which is 12.4 nm red shifted compared to the free form, while the dual emission peaks (Em1. λ_max_ 493.2 nm and Em2. λ_max_ 514.4 nm) remained the same. This improved detection was specific for *S. aureus*, since peak wavelengths for p‐HTAA incubated with *S*. Enteritidis (Ex. λ_max_ 394.4 nm, Em1. λ_max_ 493.6 nm, Em2. λ_max_ 513.2 nm) remained the same as free p‐HTAA.

To correlate our spectroscopy data with microscopy, we added LL‐2, HS‐72 and p‐HTAA to mixed samples of *S. aureus* and *S*. Enteritidis, and imaged each sample by confocal microscopy (Figure S2). In samples containing LL‐2, *S. aureus* cells were fluorescent, as expected from the spectroscopy data. Intriguingly, samples containing HS‐72 and p‐HTAA showed that while some *S. aureus* cells were fluorescent, others were not. As we do not fully understand why this is, we aim to investigate this further. None of the optotracers bound to *S*. Enteritidis, as reflected in all rod‐shaped cells being non‐fluorescent.

To comparatively evaluate the performance of optotracers with −2, −3 and −4 charges, we employed cross‐correlation analysis. When incubated with *S. aureus*, p‐HTAA showed the largest spectral shift (12.4±0.9 nm) followed by HS‐72 (4.4±0.9 nm) and p‐FTAA (2 nm) (Figure 3f, Table S2). p‐HTAA was the only pentameric optotracer showing increased total fluorescence when incubated with *S. aureus* (2.11±0.93) (Figure 3g, Table S2). Importantly, the improvement of bacterial detection was selective for *S. aureus* as incubation with *S*. Enteritidis did not result in any spectral shifts or fluorescence increase for any of the pentamers.

While the presence of negative charges is essential to maintain water solubility of the optotracers, our analysis of differently charged pentamers showed that detection of *S. aureus* can be greatly enhanced by reducing the number of negatively charged groups along the thiophene backbone. In addition, comparisons of spectra from heptameric and pentameric optotracers with approximately the same charge per thiophene ring (LL‐2, 0.86 negative charges/thiophene ring; p‐FTAA, 0.8 negative charges/thiophene ring) revealed a 6‐fold larger spectral shift for the heptamer LL‐2. Similarly, comparison of optotracers with the same total charge (h‐FTAA versus p‐FTAA) showed a 19‐fold larger shift of the heptamer compared to the pentamer. Thus, the length of the conjugated backbone as well as the amount of negatively charge groups along the backbone influence the detection of *S. aureus*.

### Role of the molecular structure for optotracer detection of *S. aureus*

**Figure 4:**
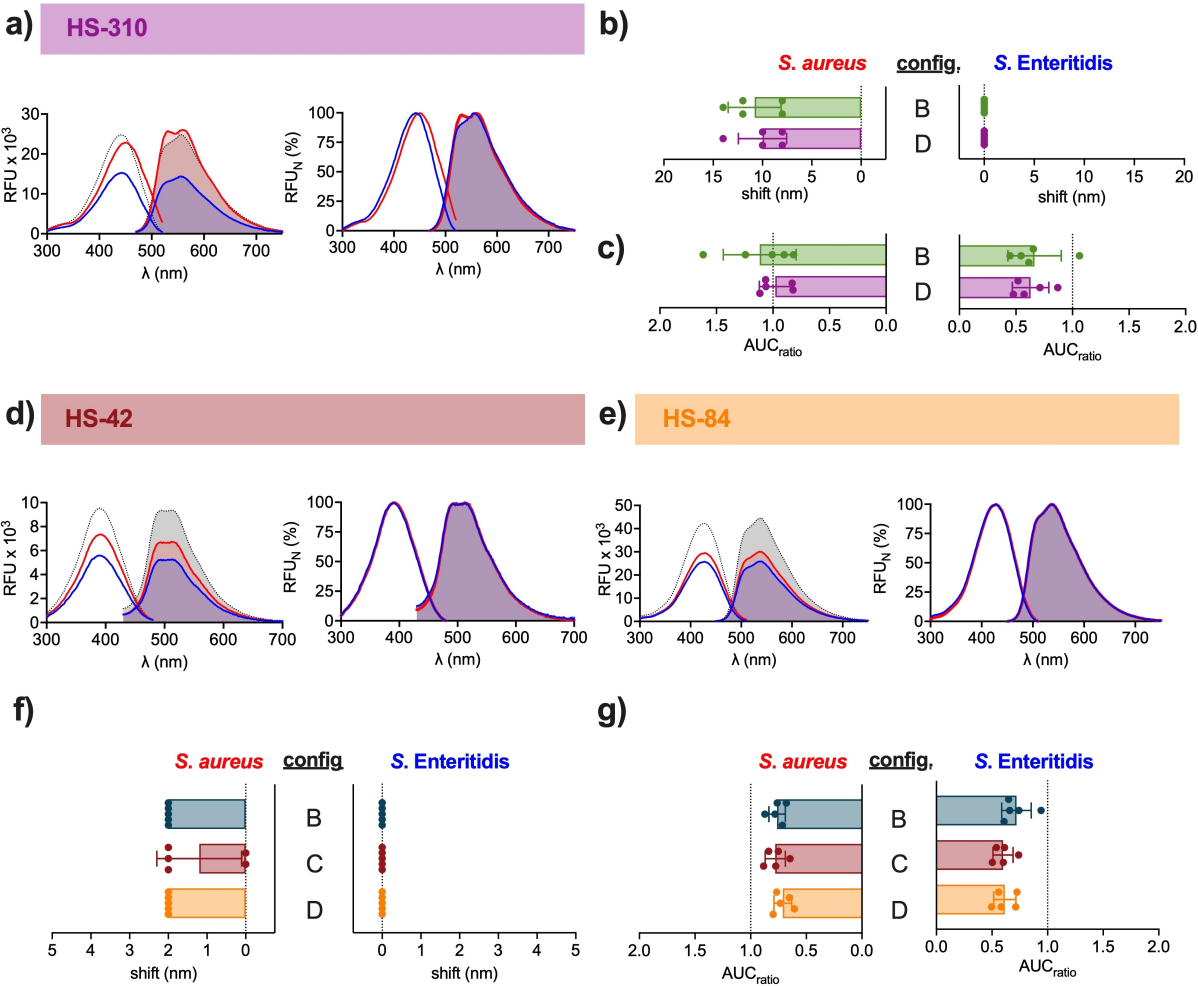
Role of charge distribution for optotracer detection of *S. aureus*. Spec‐plots (left) and normalized spec‐plots (right) of: a) HS‐310, d) HS‐42 and e) HS‐84 in free form (dotted line) and mixed with *S. aureus* (red line) or *S*. Enteritidis (blue line). Lines show mean of n=5. (b, f) Spectral shifts calculated by cross‐correlation analysis of excitation spectra of the hexamers b) hx‐FTAA (configuration B) and HS‐310 (configuration D), and the pentamers f) p‐FTAA (configuration B), HS‐42 (configuration C) and HS‐84 (configuration D) mixed with *S. aureus* or *S*. Enteritidis. The change in fluorescence intensity, calculated as the ratio of the area under curve (AUC_ratio_) of the excitation spectra of c) hx‐FTAA and HS‐310 and g) p‐FTAA, HS‐42, and HS‐84, mixed with *S. aureus* or *S*. Enteritidis, and the AUC of free optotracer. Bars in (b, c, f, g) show mean of n=5±SD, individual data points are shown as filled circles. The color‐coded names of optotracers correspond to the colour codes of bars. Optrotracers were used at 1 μM.

To determine if the distribution of negatively charged groups along the thiophene backbone influences bacterial detection, we used optotracers of the same length and total charge but different charge distribution. From a variety of previously synthesised pentameric and hexameric anionic oligothiophenes with the same charge as p‐FTAA and hx‐FTAA,[^13^, ^20^] we selected the hexamer HS‐310 (Figure 4a) and the pentamers HS‐42 (Figure 4d) and HS‐84 (Figure 4e) as complements to the panel of optotracers used to evaluate the effect of distinct charge distribution along the thiophene backbone. The hexameric optotracers HS‐310 and hx‐FTAA have four anionic groups each, which are differently spaced (Figure 1c). Peak analysis of HS‐310 (configuration D) showed 7.6 nm red‐shifted Ex. λ_max_ (449.6 nm) and a plateaued, dual‐peak emission (Em1. λ_max_ 532.4 nm and Em2. λ_max_ 560 nm) when mixed with *S*. *aureus* compared to the free form (Ex. λ_max_ 442 nm, Em. λ_max_ 556 nm) (Figure 4a, Table S1). Mixing with *S*. Enteritidis had no influence on HS‐310 spectra. Peak analysis of hx‐FTAA (configuration B) gave nearly identical spectral patterns for the free form and when mixed with *S. aureus* (Figure 2c). Cross‐correlation analysis revealed a circa 10 nm shift for both optotracers mixed with *S. aureus*, but not for *S*. Enteritidis (Figure 4b). Moreover, mixing of the hexamers with bacteria did not cause any change in fluorescence intensity (Figure 4c, Table S2). This suggests that the minor alteration in the distribution of charged groups does not influence the spectral properties of free HS‐310 and hx‐FTAA or their interaction with *S. aureus* and *S*. Enteritidis.

We next analysed the pentameric optotracers p‐FTAA (configuration B), HS‐42 (configuration C), and HS‐84 (configuration D) (Figure 1c). Peak analysis of data presented in (Figure 2b) showed no shifts of p‐FTAA irrespective of bacterial species. Peak analysis of HS‐42 showed Ex. λ_max_ 390 nm and a plateaued, dual‐peak emission (Em1. λ_max_ 493.6 nm, Em2. λ_max_ 512 nm) of the free form, which remained upon mixing with *S. aureus* (Ex. λ_max_ 390.8 nm, Em1. λ_max_ 494.4 nm, Em2. λ_max_ 513.6 nm) and *S*. Enteritidis (Ex. λ_max_ 390 nm, Em1. λ_max_ 494 nm, Em2. λ_max_ 512 nm) (Figure 4d). An analogous pattern was observed for HS‐84 in which Ex. λ_max_ (426 nm) and Em. λ_max_ (537.6 nm) of the free form remained upon mixing with *S. aureus* (Ex. λ_max_ 426.8 nm, Em λ_max_ 536.8 nm) and *S*. Enteritidis (Ex. λ_max_ 427.2 nm, Em. λ_max_ 536.4 nm) (Figure 4e). Cross correlation analysis showed 2 nm shifts for p‐FTAA and HS‐84 mixed with *S. aureus*, as well as in 3 out of 5 comparisons for HS‐42 (Figure 4f, Table S2). None of the optotracers showed any shifts in the presence of *S*. Enteritidis. Quantification of fluorescence intensity revealed decreased fluorescence for optotracers mixed with bacteria compared to their free form, and no difference were observed between the pentamers (Figure 4g, Table S2). Collectively, this shows that the placing of charged groups along the thiophene backbone has no influence on their ability to detect *S. aureus* or *S*. Enteritidis.

### Fluorescence microscopy demonstrates optotracer binding to the cell wall of *S. aureus*

**Figure 5:**
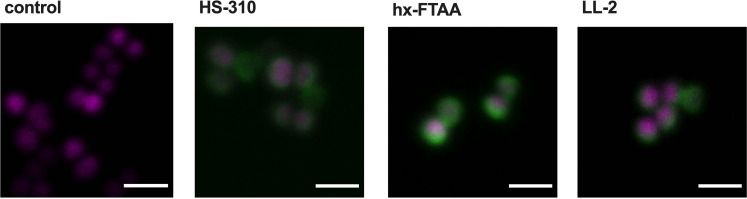
Optotracers HS‐310, hx‐FTAA and LL‐2 bind to the cell wall of *S. aureus*. Confocal fluorescence microscopy of ARD348 (*S. aureus* expressing the red fluorescent protein FP650) in the absence (control) and the presence of HS‐310, hx‐FTAA and LL‐2. Fluorescence from bound optotracers (green) was recorded using 488 nm excitation laser, with emission collected below 625 nm. FP650 (magenta) was recorded using 561 nm excitation laser, with emission collected above 650 nm. Representative images of n=3 are shown. Scale bar=2 μm. Images were processed as described in Experimental Section. The raw images are shown in Figure S3.

Our first demonstration of optotracer detection of *S. aureus* showed that the fluorescence signal originated from optotracers bound to the bacterial cell wall.^[14]^ To investigate if the same principle applies to the present study, we selected optotracers showing the largest and most consistent spectral shifts (Table S2). This identified the hexamers HS‐310 and hx‐FTAA, and the heptamer LL‐2 as relevant candidates. To perform confocal fluorescence microscopy, we mixed the strain ARD348, an isogenic *S. aureus* with cytoplasmic expression of the red fluorescent protein FP650, with each of the three optotracers and imaged cells under the 100× objective. In all samples, including the control, red fluorescence from FP650 clearly visualized the bacterial cytoplasm (Figure 5). In samples mixed with HS‐310, hx‐FTAA and LL‐2, the periphery of each bacterial cell was clearly delineated by green fluorescence, which was absent from the control. Collectively, this confirmed that HS‐310, hx‐FTAA and LL‐2 predominantly bind to the cell wall, thereby corroborating our previously reported finding.

### Introducing D‐A‐D motif(s) for optimized detection of bacteria

**Figure 6:**
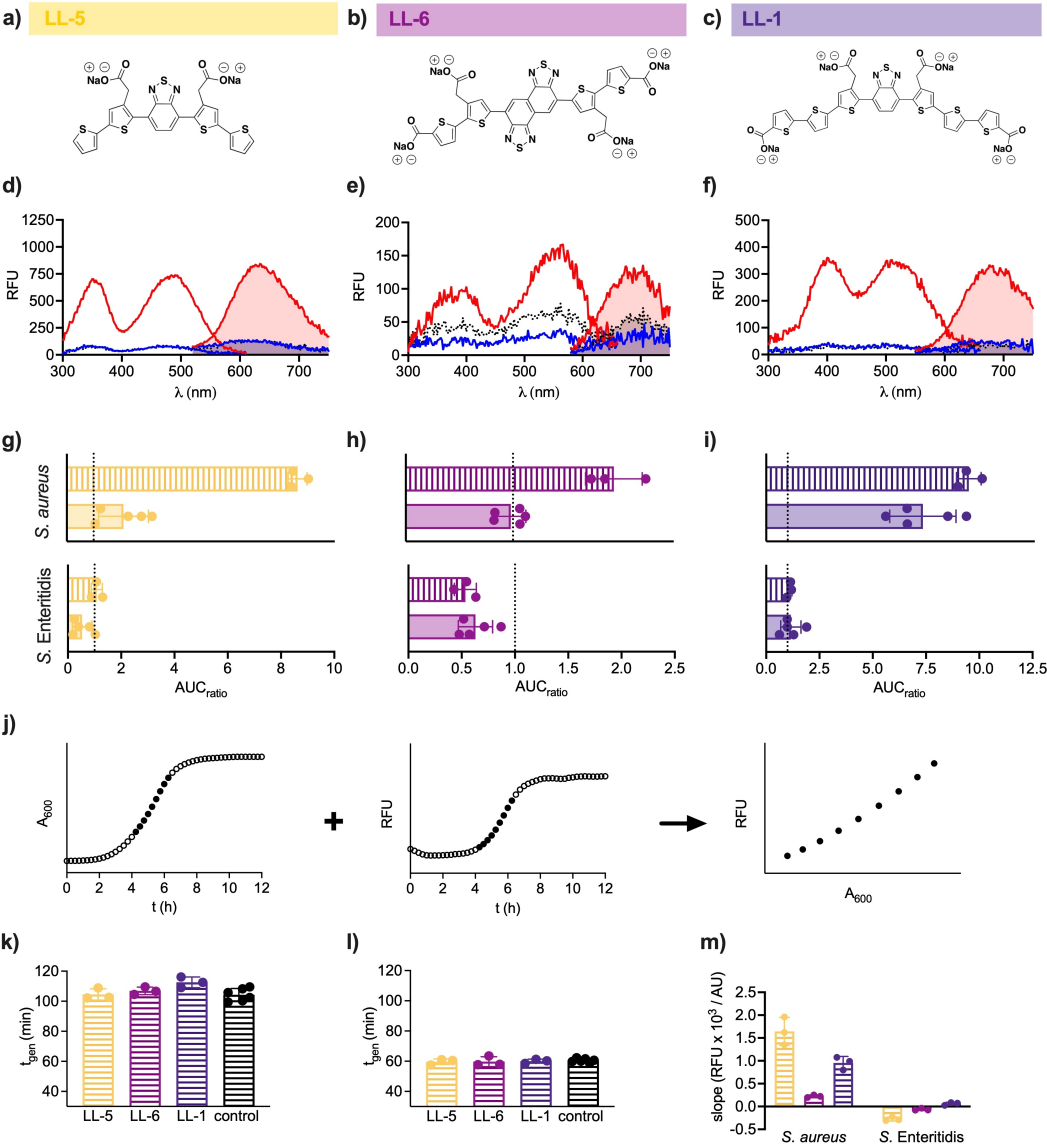
Optotracers with D‐A‐D motifs enable real‐time optotracing of bacterial growth. Structures of a) LL‐5, b) LL‐6 and c) LL‐1. Spec‐plot showing d) LL‐5, e) LL‐6 and f) LL‐1 in free form (dotted line) and mixed with *S. aureus* (red line) or *S*. Enteritidis (blue line). Lines show mean of n=3. g–i) The change in fluorescence intensity calculated as the ratio of the area under curve (AUC) of the excitation spectra of g) p‐HTAA (solid) and LL‐5 (patterned), h) HS‐310 (solid) and LL‐6 (patterned), and i) h‐FTAA (solid) and LL‐1 (patterned) mixed with *S. aureus* or *S*. Enteritidis (AUC_sample_), and the AUC of free optotracer (AUC_optotracer_). Bars show mean±SD of n≥3, individual data points are shown as filled circles. j) Schematic showing how the exponential growth phase from absorbance and fluorescence recordings are combined to enable real‐time optotracing recording of bacterial growth. k, l) Generation time of k) *S. aureus* and l) *S*. Enteritidis growing in medium supplemented with the D‐A‐D optotracers LL‐5, LL‐6, LL‐1. Control=no added optotracer. m) The slopes (RFU×10^3^/AU) generated by real‐time optotracing recording of growth of *S. aureus* and *S*. Enteritidis in medium supplemented with LL‐5 (yellow), LL‐6 (magenta) and LL‐1 (violet). k–m) Bars show mean±SD of n=3, filled circles show individual data points. Optrotracers were used at 1 μM.

Having identified the structural determinants governing *S. aureus* detection, we next optimised the photophysical properties of optotracers such that their binding is reported by increased fluorescence intensity rather than spectral shifts. We thus modified the thiophene backbone to include a central D‐A‐D motif that is known to enhance binding‐induced emission intensity[^14^, ^18^] To synthesize the D‐A‐D variants of the pentamer, hexamer, and heptamer, we replaced the central thiophene ring in p‐HTAA and h‐FTAA with the electron acceptor benzothiadiazole, while the central rings in HS‐310 were replaced by naphthobisthiadiazole. In the resulting optotracers LL‐5, LL‐6 and LL‐1 (Figure 6a–c), the electron rich thiophene units act as donors and the electron‐withdrawing BTD motif(s) as acceptor(s). LL‐5 and LL‐6 were synthesized as outlined in Supplementary Scheme 1, while the synthesis of LL‐1 has been previously reported.^[19]^ Briefly, the synthetic methodologies involved bromination with *N*‐bromosuccinimide (NBS), palladium catalysed cross‐couplings through Suzuki‐Miyaura reactions, and carboxylic acid protecting group chemistry with methyl esters. When analysing the excitation and emission spectra of LL‐5, LL‐6 and LL‐1 in free form, the fluorescence intensities were very low (Figure 6d–f). In contrast, a prominent increase in fluorescence intensities were shown from all D‐A‐D optotracers mixed with *S. aureus*, with LL‐5 showing the highest absolute fluorescence. A signal similarly low as the free form was observed from D‐A‐D optotracers mixed with *S*. Enteritidis. Taken together, this demonstrated that the D‐A‐D motif enabled the use of increased fluorescence intensity as main indicator of optotracers binding to the bacterial cell.

To analyse the performance of D‐A‐D optotracers to their oligothiophene counterparts, we calculated the relative fluorescence intensity by comparing the area under curve (AUC) of the optotracer with (AUC_sample_) and without (AUC_optotracer_) bacteria. When mixed with *S. aureus*, the AUC_ratio_ of the pentamer LL‐5 (8.6) was >4 times that of p‐HTAA (2.1) (Figure 6g), the hexamer LL‐6 (1.94) was almost twice as high as HS‐310 (0.98) (Figure 6h), and the heptamer LL‐1 (9.43) was circa 30 % higher than h‐FTAA (7.29) (Figure 6i). Thus, LL‐1 showed the greatest AUC_ratio_ whereas the largest difference between the D‐A‐D optotracer and its oligothiophene counterpart was observed for LL‐5/p‐HTAA. When mixed with *S*. Enteritidis, no fluorescence increase was found for any of the D‐A‐D optotracers (Figure 6g–i). Collectively, while not influencing the optotracers’ binding selectivity, the D‐A‐D motif affords an on‐switch of fluorescence when optotracers bind *S. aureus* cells.

Next, we tested whether the presence of D‐A‐D motifs in the thiophene backbone influences the utility of LL‐5, LL‐6, and LL‐1 as real‐time tracers of bacterial growth, using high‐throughput real‐time optotracing.^[14]^ In this recently developed method, schematically shown in Figure 6j, A_600_ and the fluorescence signal were measured every 15 min during growth in buffered medium supplemented with D‐A‐D optotracers. The exponential phase was determined, and the generation time was calculated. Also, for each time point collected in the exponential phase, the fluorescence signal (RFU) was plotted against the corresponding A_600_ (AU), from which a slope value (RFU/AU) was determined by linear fitting. Calculation of the generation time for *S. aureus* (Figure 6k) and *S*. Enteritidis (Figure 6l) showed no differences between cultures grown in the absence and presence of optotracers, suggesting that these molecules are non‐toxic and therefore suitable for real‐time experiments. When analysing the RFU/AU, the positive slope values of LL‐5, LL‐6 and LL‐1 in the presence of *S. aureus* demonstrate the ability of all three optotracers to bind *S. aureus* cells (Figure 6m). In contrast, slopes at or below 0 were found in cultures of *S*. Enteritidis, showing a lack of optotracer binding. Taken together, these data showed that addition of the D‐A‐D motif to p‐HTAA, HS‐310, and h‐FTAA did not interfere with the ability of optotracers to distinguish between the bacterial species. The modification did, however, add the essential photophysical properties required to enable real‐time monitoring of bacterial growth.

## Conclusion

By employing a selection of chemically well‐defined optotracers, we identified the structural properties providing optimal detection of the important bacterial pathogen *S*. *aureus* while preserving selectivity towards Gram‐negative *S*. Enteritidis. We demonstrated that the length of the conjugated backbone and the number of charged groups, but not their distribution, are important for optotracer detection of *S. aureus*, as reflected by larger shifts of excitation spectra. This suggests that the thiophene moieties promote attractive hydrophobic and aromatic interactions, while negatively charged groups lead to repulsion necessary to tune the selectivity of detection.

Rapid, robust, and objective determination of spectral shifts of optotracers bound to bacterial cells was achieved by complementing peak excitation analysis, which is based on fluorescence at a single wavelength, with cross‐correlation analysis that takes fluorescence information at all wavelengths of the recorded spectrum into account. By implementing a method rooted in the field of signal processing, we developed a robust analytical technique for optotracing detection that facilitates spectral analytics for trained and untrained users. To further simplify the data analysis, we analysed optotracer binding to bacteria based on total fluorescence intensity. By calculating area under the curve of aligned excitation spectra, the fluorescence ratio of bound versus free optotracers was obtained. This analytical scheme proved specifically useful for optotracers containing the D‐A‐D motif. Their on‐switch of fluorescence upon binding to *S. aureus* enabled tracing of bacterial growth in live cultures, and we showed that the slopes generated when plotting fluorescence versus absorbance at a given time point enabled real‐time monitoring of bacterial growth. Having laid the foundation for optotracing analytics of bacterial cultivation, this work enables future design of superior optotracers for a variety of applications in microbiology research and infection diagnostics.

## Experimental Section

**Chemicals**: Phosphate buffer saline (PBS) pH 7.4 (Medicago, Sweden) was prepared in ultrapure water and autoclaved before use. Optotracers, synthesized as previously reported[^7^, ^9^, ^10^, ^13^, ^19^, ^20^] and as described in Supplementary Information, were kept at 1.5 mM in ultrapure water and used at 1 μM unless stated differently.

**Bacterial strains, plasmids, and growth conditions**: *Staphylococcus aureus* (*S. aureus*) 8325‐4^[21]^ and *Salmonella* Enteritidis (*S*. Enteritidis) 3934^[22]^ were maintained as stocks in −80 °C. Strain ARD348 was created by introducing the plasmid pSFRFPS1,^[23]^ which encodes the red fluorescent protein FP650, to *S*. *aureus* 8325‐4 using medium supplemented with trimethoprim (25 μg/mL). To prepare overnight cultures, a single colony from strains grown on Tryptic Soy agar (Sigma‐Aldrich, Sweden) was used to inoculate 5–10 mL Tryptic Soy Broth (TSB, Sigma‐Aldrich, Sweden). Bacterial cultures were incubated at 37 °C under shaking (160 rpm) conditions overnight.

**Fluorescence spectroscopy**: Excitation and emission spectra were recorded in a Synergy™ MX plate reader (BioTek, USA). Spectra of the free form of optotracers were recorded using 1 μM optotracer in PBS. Aliquots (200 μL) transferred to flat bottom transparent 96 well plates (Sarstedt, Germany) in technical quadruplicates were recorded in the plate reader. Spectra from optotracers incubated with bacteria were obtained from overnight cultures of *S. aureus* and *S*. Enteritidis that had been washed twice and resuspended in PBS to an optical density (OD_600_) of circa 2.0. Then, each optotracer (1 μM) was added to bacterial suspensions, and spectra from 200 μL aliquots transferred to flat bottom transparent 96 well plates (Sarstedt, Germany) in technical duplicates were recorded in the plate reader. In the plate reader, excitation and emission spectra were recorded with 9 nm margin, 2 nm step size and 100 % sensitivity. Settings applied for each optotracer were:

**q‐FTAA**: λ_Ex_ 300–480 nm at λ_Em_ 520 nm, λ_Em_ 410–700 nm at λ_Ex_ 390 nm

**p‐HTAA**: λ_Ex_ 300–480 nm at λ_Em_ 515 nm, λ_Em_ 430–700 nm at λ_Ex_ 398 nm

**p‐FTAA**: λ_Ex_ 300–510 nm at λ_Em_ 543 nm, λ_Em_ 440–750 nm at λ_Ex_ 414 nm

**HS‐84**: λ_Ex_ 300–510 nm at λ_Em_ 537 nm, λ_Em_ 450–750 nm at λ_Ex_ 427 nm

**HS‐72**: λ_Ex_ 300–510 nm at λ_Em_ 541 nm, λ_Em_ 450–750 nm at λ_Ex_ 428 nm

**HS‐42**: λ_Ex_ 300–480 nm at λ_Em_ 513 nm, λ_Em_ 430–700 nm at λ_Ex_ 391 nm

**HS‐310**: λ_Ex_ 300–520 nm at λ_Em_ 555 nm, λ_Em_ 470–750 nm at λ_Ex_ 443 nm

**hx‐FTAA**: λ_Ex_ 300–530 nm at λ_Em_ 556 nm, λ_Em_ 470–750 nm at λ_Ex_ 444 nm

**h‐FTAA**: λ_Ex_ 300–550 nm at λ_Em_ 577 nm, λ_Em_ 460–750 nm at λ_Ex_ 435 nm

**LL‐1**: λ_Ex_ 300–660 nm at λ_Em_ 685 nm, λ_Em_ 550–750 nm at λ_Ex_ 520 nm

**LL‐2**: λ_Ex_ 300–550 nm at λ_Em_ 576 nm, λ_Em_ 470–740 nm at λ_Ex_ 437 nm.

**LL‐5**: λ_Ex_ 300–610 nm at λ_Em_ 640 nm, λ_Em_ 520–750 nm at λ_Ex_ 488 nm

**LL‐6**: λ_Ex_ 300–660 nm at λ_Em_ 690 nm, λ_Em_ 580–750 nm at λ_Ex_ 556 nm

**Fluorescence microscopy**: Bacterial samples with and without optotracers were prepared as described for spectroscopy. For mixed samples, resuspensions of *S. aureus* and *S*. Enteritidis in PBS were mixed 1 : 1, optotracers were then added and the sample was immediately mounted on a microscope slide with a coverslip sealed by nail polish. Confocal fluorescence microscopy of the mixed samples was performed using an Olympus FV1000 with 60× water objective. The 405 nm excitation laser was used for q‐FTAA and p‐FTAA, 473 nm excitation laser was used for hx‐FTAA and h‐FTAA. Emission from all four optotracers was collected at 490–590 nm. Imaging of ARD348 (*S*. *aureus* expressing FP650) mixed with hx‐FTAA, HS‐310 and LL‐2 was performed on a Zeiss LSM800 confocal microscope using 100× oil immersion objective. Optotracer detection was achieved using the 488 nm excitation laser, with emission collected <625 nm. Detection of the red fluorescent protein FP650 was achieved using the 561 nm excitation laser, with emission collected >650 nm. Representative images from biological replicates of n≥3 are shown if not indicated otherwise.

**Image processing**: Images were processed using the FIJI^[24]^ imaging software. Figure 5 shows images processed using *Despeckle* function and applying the *Smooth* function.

**Data analysis**: Automated data analysis and visualization was performed with Python programming language using SciPy,^[25]^ Plotly,^[26]^ NumPy,^[27]^ Pandas^[28]^ and Matplotlib^[29]^ packages. Technical multiplicates from each biological replicate were averaged. To determine peaks, the data were filtered with Savitzky‐Golay digital filter, using window length 11 and polynomial order of 3. Peaks were identified using scipy.signal.find_peaks, a function that identifies local maxima by comparing neighbouring values. When necessary, the data were manually checked. For cross‐correlation analysis, input vectors were normalized to unit length. When cross‐correlating two signals, the formula essentially slides one of the signals along the x‐axis, calculating the integral of their product at each position. The maximum value of the integral defines the position where the two signals match best. To quantify the fluorescence increase, data were first aligned such that a maximum overlap was achieved. The integrated fluorescence, the so‐called area under curve (AUC), was calculated from the aligned spectra using the trapezoidal rule. To determine the relative fluorescence increase, the integrated fluorescence of the sample was divided by the integrated fluorescence of the control. Prism 8 (GraphPad Software Inc, USA) was used to plot the data.

## Supporting information

As a service to our authors and readers, this journal provides supporting information supplied by the authors. Such materials are peer reviewed and may be re‐organized for online delivery, but are not copy‐edited or typeset. Technical support issues arising from supporting information (other than missing files) should be addressed to the authors.

Supporting Information
