# Supplementary material for: Structural Properties Dictating Selective Optotracer Detection of Staphylococcus aureus
Source: Chembiochem. 2022 Apr 1;23(11):e202100684. doi: 10.1002/cbic.202100684 (PMC9400997; doi:10.1002/cbic.202100684)
Supplement: Supplementary file 1 — Supporting Information [file CBIC-23-0-s001.pdf]

ChemBioChem

Supporting Information

Structural Properties Dictating Selective Optotracer Detection of *Staphylococcus aureus*

Karen Butina, Linda Lantz, Ferdinand X. Choong, Ana Tomac, Hamid Shirani, Susanne Löffler, K. Peter R. Nilsson, and Agneta Richter-Dahlfors*

Author Contributions

K.B. Conceptualization:Lead; Data curation:Lead; Formal analysis:Lead; Investigation:Lead; Methodology:Lead; Writing – original draft:Lead; Writing – review & editing:Lead
L.L. Formal analysis:Supporting; Investigation:Supporting
F.C. Conceptualization:Supporting; Formal analysis:Supporting; Visualization:Supporting; Writing – review & editing:-Supporting
A.T. Methodology:Supporting; Software:Supporting
H.S. Formal analysis:Supporting; Investigation:Supporting
S.L. Software:Supporting; Supervision:Supporting; Writing – review & editing:Supporting
K.N. Conceptualization:Lead; Resources:Supporting; Supervision:Supporting; Writing – review & editing:Supporting
A.R.-D. Conceptualization:Lead; Formal analysis:Supporting; Funding acquisition:Lead; Project administration:Lead; Resources:Lead; Supervision:Lead; Writing – original draft:Supporting; Writing – review & editing:Lead

Confocal microscopy of mixed *S. aureus* and *S.* Enteritidis using optotracing

Figure S1 a) Confocal micrographs of mixed *S. aureus* and *S.* Enteritidis with added optotracers q-FTAA, p-FTAA, hx-FTAA and h-FTAA at 1 µM. For q-FTAA, no signal from any of the bacterial species was observed, as expected from the fluorescence spectroscopy. For p-FTAA, no signal was observed for any of the bacterial species, showing that the 2 nm spectral shift observed for *S. aureus* by spectroscopy is insufficient to obtain a visual signal under the microscope. In the presence of hx-FTAA, *S. aureus* cells were brightly fluorescent, while *S.* Enteritidis remained unstained. In the presence of h-FTAA, *S. aureus* cells showed fluorescence, which was slightly weaker than that from hx-FTAA. *S.* Enteritidis remained unstained, corroborating the previous observation that a spectral shift of circa 3 nm, observed for h-FTAA mixed with *S.* Enteritidis, is insufficient to obtain a visual signal under the microscope.

Figure S1b) We previously reported weak fluorescence from HS-84 (pentameric optotracer similar to p-FTAA) when added at 5 µM to *S. aureus* (Butina *et al.* Optotracing for selective fluorescence-based detection, visualization and quantification of live *S. aureus* in real-time. *npj Biofilms Microbiomes* **6**, (2020)). This urged us to analyse if the lack of signal from q-FTAA and p-FTAA may be concentration dependent. Using 5 µM q-FTAA and p-FTAA, confocal microscopy showed weak fluorescence of *S. aureus* for both optotracers. This corroborated our previous data on HS-84. The weak signal at the 5-fold increased concentration can be explained by *i)* Low level binding occurs but the signal is below threshold at 1 µM; *ii)* Supramolecular structures such as micelles form at higher concentrations leading to a different binding mode.

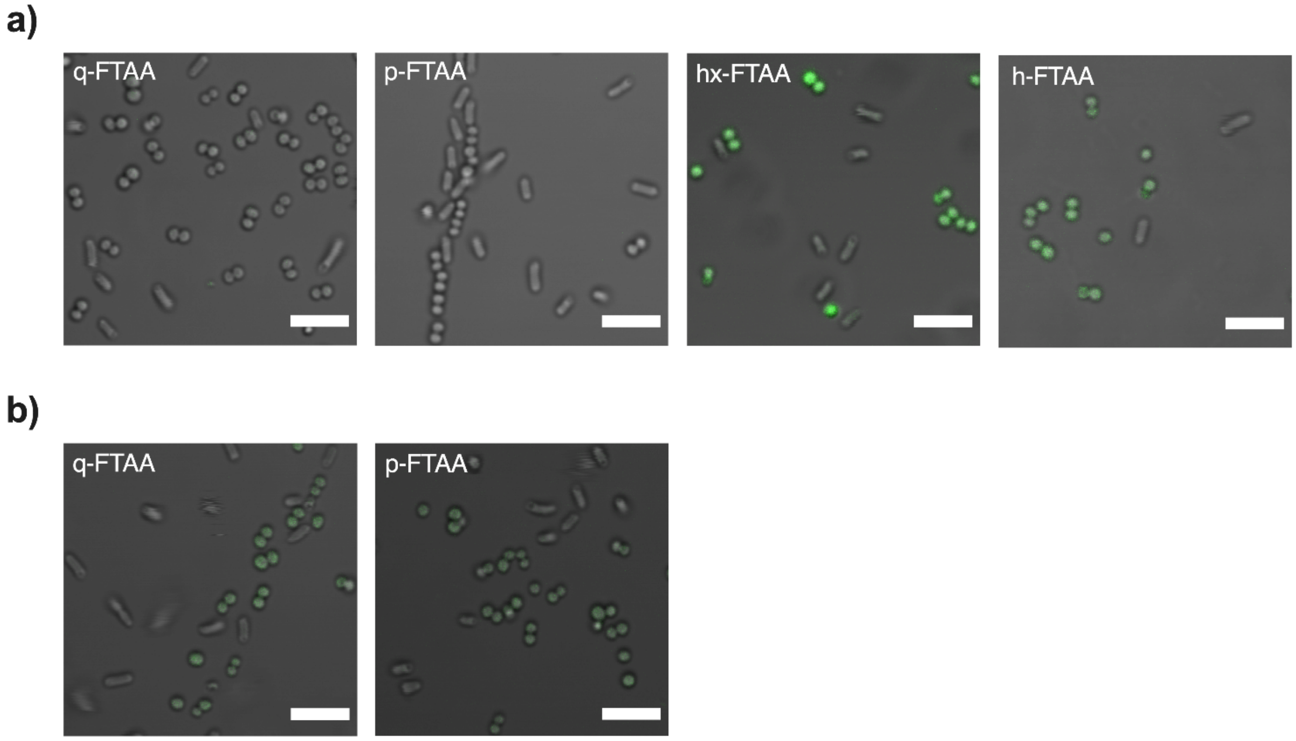

Figure S1. Selective detection of *S. aureus* in samples mixed with *S.* Enteritidis with optotracers q-FTAA, p-FTAA, hx-FTAA, h-FTAA. Merged translight and confocal (pseudocoloured green) images of mixed samples containing *S. aureus* (gram-positive, coccoid) and *S.* Enteritidis (gram-negative, rod-shaped) with (**a**) 1 μM optotracers (q-FTAA, p-FTAA, hx-FTAA, h-FTAA), (**b**) 5 μM optotracers (q-FTAA, p-FTAA). Scale bar = 5 μm. Representative images from biological replicates of n ≥ 3 are shown.

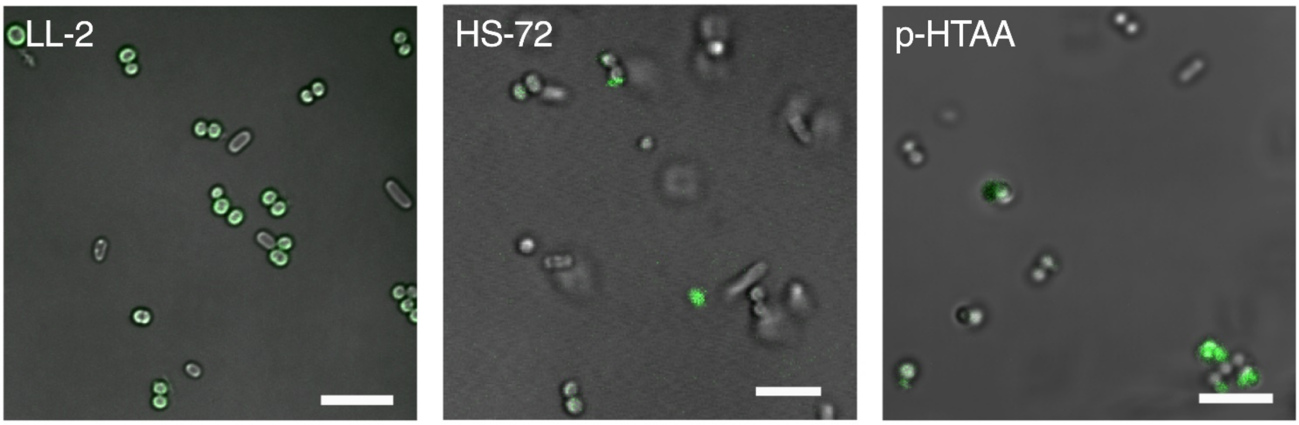

Figure S2. Selective detection of *S. aureus* in samples mixed with *S.* Enteritidis with optotracers LL-2, HS-72 and p-HTAA. Merged translight and confocal (pseudocoloured green) images of mixed samples containing *S. aureus* (gram-positive, coccoid) and *S.* Enteritidis (gram-negative, rod-shaped) with 1 μM optotracers (LL-2, HS-72, p-HTAA). Scale bar = 5 μm. Representative images from biological replicates of n = 2 are shown.

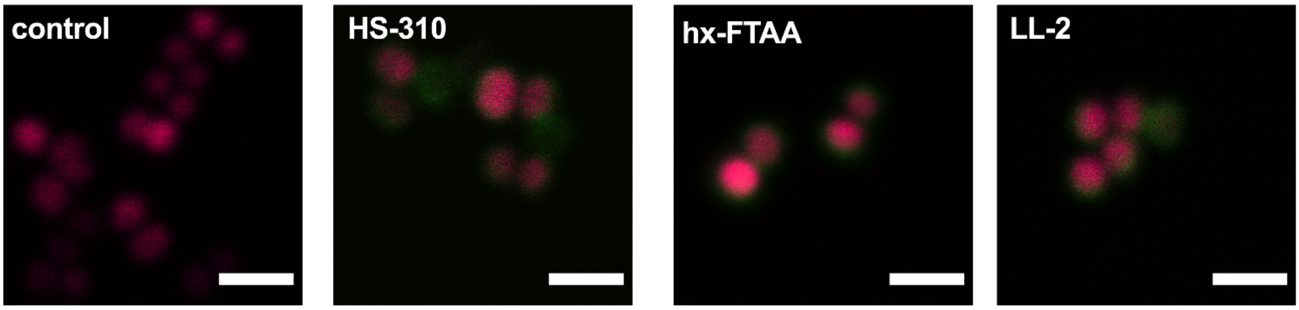

Figure S3. Unprocessed images showing binding of optotracers HS-310, hx-FTAA and LL-2 to the cell wall of *S. aureus*. Confocal fluorescence microscopy of ARD348 (*S. aureus* expressing the red fluorescent protein FP650) in the absence (control) and the presence of HS-310, hx-FTAA and LL-2. Fluorescence from bound optotracers (green) was recorded using 488 nm excitation laser, with emission collected below 625 nm. FP650 (magenta) was recorded using 561 nm excitation laser, with emission collected above 650 nm. Representative images of n = 3 are shown. The same images, processed using ImageJ software are shown in **Figure 5** of the main manuscript. Scale bar = 2 μm.

Table S1 and **Table S2**

Table S1. Peak excitation and emission wavelengths of optotracers in the absence and presence of bacteria

	**Optotracer**			**Optotracer + *S. aureus***			**Optotracer + *S.* Enteritidis**		
	mean ± SD (nm)			mean ± SD (nm)			mean ± SD (nm)		
	**Ex. λ_{max}**	**Em1. λ_{max}**	**Em2. λ_{max}**	**Ex. λ_{max}**	**Em1. λ_{max}**	**Em2. λ_{max}**	**Ex. λ_{max}**	**Em1. λ_{max}**	**Em2. λ_{max}**
**q-FTAA**	390 ± 0	520.4 ± 0.9		390 ± 0	519.6 ± 1.7		390 ± 0	520.4 ± 1.7	
**p-FTAA**	414.4 ± 0.9	543.6 ± 0.9		416 ± 0	542.8 ± 1.1		414 ± 0	544 ± 1.4	
**hx-FTAA**	442 ± 0	556 ± 0		450.4 ± 2.6	532.4 ± 1.7	560.4 ± 0.9	443.2 ± 1.1	556 ± 0	
**h-FTAA**	434.8 ± 1.8	553.6 ± 1.7		478 ± 1.4	550.4 ± 0.9		435.2 ± 2.7	545.6 ± 3.4	
**LL-2**	436.4 ± 0.9	573.6 ± 1.7		447.2 ± 2.3	558.4 ± 5.5	574.8 ± 3	436.4 ± 1.7	573.6 ± 1.7	
**p-HTAA**	395.6 ± 0.9	494.4 ± 1.4	514.8 ± 1.1	408 ± 2.4	493.2 ± 1.1	514.4 ± 1.7	394.4 ± 2.6	493.6 ± 1.7	513.2 ± 1.1
**HS-72**	425.6 ± 0.9	540 ± 0		428.8 ± 1.8	540.4 ± 0.9		425.2 ± 1.1	540 ± 0	
**HS-310**	442 ± 0	556 ± 0		449.6 ± 3	532.4 ± 1.7	560 ± 1.4	442.8 ± 1.1	556.4 ± 0.9	
**HS-42**	390 ± 0	493.6 ± 0.9	512 ± 0	390.8 ± 1.8	494.4 ± 0.5	513.6 ± 0.9	390 ± 1.4	494 ± 1.4	512 ± 0
**HS-84**	426 ± 0	537.6 ± 0.9		426.8 ± 1.1	536.8 ± 1.1		427.2 ± 1.1	536.4 ± 0.9	

Table S2. Structural properties, Spectral shift and Relative fluorescence increase of optotracers in the presence of *S. aureus* and *S.* Enteritidis

				**Spectral shift**				**Relative fluorescence increase**			
				S. aureus		***S.* Enteritidis**		***S. aureus***		***S.* Enteritidis**	
**Name**	**Length**	**Charge**	**Configu-ration**[a]	mean ± SD (nm)	CV[b] (%)	mean ± SD (nm)	CV (%)	mean ± SD	CV (%)	mean ± SD	CV (%)
**q-FTAA**	4	-3	B	0 ± 0	NA	0 ± 0	NA	0.65 ± 0.08	12.3	0.64 ± 0.09	14.1
**p-FTAA**	5	-4	B	2 ± 0	0	0 ± 0	NA	0.76 ± 0.07	9.2	0.72 ± 0.13	18
**hx-FTAA**	6	-4	B	10.8 ± 2.7	25	0 ± 0	NA	1.12 ± 0.32	28.6	0.66 ± 0.24	36.4
**h-FTAA**	7	-4	B	38 ± 1.4	3.7	3.6 ± 1.7	47	7.30 ± 1.54	21.1	1.16 ± 0.48	41.4
**LL-2**	7	-6	E	13.2 ± 2.3	17.4	0 ± 0	NA	1.04 ± 0.08	7.7	0.69 ± 0.17	24.6
**p-HTAA**	5	-2	B	12.4 ± 0.9	7.3	0.4 ± 1.7	425	2.11 ± 0.93	44.1	0.53 ± 0.37	69.8
**HS-72**	5	-3	A	4.4 ± 0.9	20.5	0 ± 0	NA	0.86 ± 0.16	18.6	0.65 ± 0.10	15.4
**HS-310**	6	-4	D	10 ± 2.5	25	0 ± 0	NA	0.98 ± 0.14	14.3	0.63 ±0.16	25.4
**HS-42**	5	-4	C	1.2 ± 1.1	91.7	0 ± 0	NA	0.78 ± 0.09	11.5	0.60 ± 0.09	15
**HS-84**	5	-4	D	2 ± 0	0	0 ± 0	NA	0.71 ± 0.08	11.3	0.62 ± 0.10	16.1

[a] Configuration: Distribution of anionic groups
A = 1acetate on the central thiophene unit and 2 α-terminal carboxylates
B = head-to-head acetate groups and 2 α-terminal carboxylates
C = head-to-head acetate groups in the β-position of thiophenes 2,4 and 1,5
D = tail-to-tail acetate groups and 2 α-terminal carboxylates
E = head-to-head and tail-to-tail acetate groups to the 3 central thiophene moieties; 2 α-terminal carboxylates.

[b] CV = coefficient of variation (SD/mean)

Experimental Details

Synthesis of optotracers

The synthesis of q-FTAA, p-FTAA, hx-FTAA, h-FTAA, p-HTAA, HS-42, HS-84, HS-72, HS-310, LL-1 and LL-2 has been published elsewhere.[1-6] LL-5 and LL-6 were synthesized according to the following procedures.

General methods

Reagents and solvents were purchased from Sigma Aldrich, Merck KGaA. Flash column chromatography was performed using silica gel (60 Å, 0.040-0.063 mm, 230-400 mesh particle size, Sigma Aldrich, Merck KGaA). NMR spectra were recorded on Varian instrument (^{1}H 500 MHz, ^{13}C 126 MHz, Varian Inc., Santa Clara, CA, USA) and chemical shifts were designated on the ppm scale using residual undeuterated solvent peak as reference. Analytical thin-layer chromatography (TLC) was performed using silica gel 60 F_{254} glass plates (Supelco / Sigma Aldrich, Merck KGaA), developed in an appropriate mobile phase, visualized by UV-light λ=254 nm and 366 nm, stained with an ethanolic *p*-anisaldehyde – sulfuric acid solution (ethanol/ sulfuric acid/ *p*-anisaldehyde/ acetic acid 90:3:2:1) followed by heating for visualization.

Analytical high-performance liquid chromatography (HPLC) was performed on a Waters system comprising a Waters 1525 gradient pump, 2998 Photodiode Array Detector, 2424 Evaporative Light Scattering Detector, SQD 2 Mass Detector and a Waters XBridge™ C18 column (4.6 × 50 mm, 3.5 μm). Flow rate 1 mL/min. Eluent A: 95:5 H_2O/ acetonitrile with NH_4OAC (10 mM). Eluent B: 90:10 acetonitrile/ H_2O with NH_4OAc (10 mM).

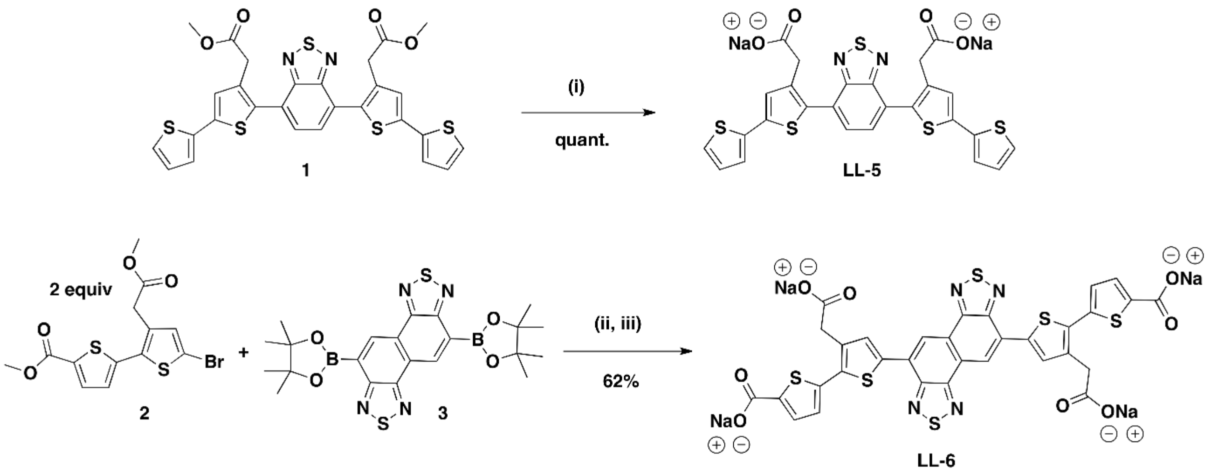

Scheme 1. Synthesis of LL-5 and LL-6. Reagents and conditions: i) NaOH (1M, aq.), 1,4-dioxane, H_2O, 35°C, 24h; (ii) PEPPSI™-*I*Pr, Cs_2CO_3, 1,4-dioxane, 75°C, 3h; (iii) NaOH, methanol/CH_2Cl_2 (1:10), r.t., 24h.

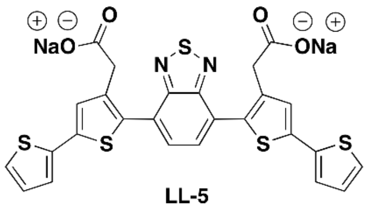

LL-5

Sodium 2,2'-(5,5''-(benzo[*c*][1,2,5]thiadiazole-4,7-diyl)bis([2,2'-bithiophene]-5,4-diyl))diacetate

To a solution of methylester **1**[5] (0.017 g, 0.028 mmol) in 1,4-dioxane (0.7 mL) was added 1M NaOH (aq.) (83.8 µL, 0.0838 mmol) dropwise. The reaction was stirred at 35°C for 2h, during this time, H_2O (1 mL) was added portionwise to the solution. The reaction was diluted with H_2O and lyophilized to give the sodium salt of LL-5 (0.0186 g, quant.) as a tomato red solid. The NMR spectrum can be found at the end of this section.

^{1}H NMR (500 MHz, $(CD_3)_2SO$) δ 8.43 (s, 2H), 7.51 (dd, *J* = 4.9 Hz, 0.98 Hz, 2H), 7.34–7.33 (m, *J* = 3.9 Hz, 0.97 Hz, 4H), 7.11 (dd, *J* = 4.9 Hz, 3.9 Hz, 2H), 3.60 (s, 4H, water peak overlapping). ^{13}C NMR (126 MHz, $(CD_3)_2SO$) δ 173.3, 153.1, 140.2, 136.8, 135.8, 130.9, 129.7, 128.4, 127.7, 125.5, 125.4, 123.7, 40.1(overlapped). HPLC-MS (ESI): *m/z* calcd for $C_{36}H_{20}N_2O_8S_7$ $(M+H)^+$: 832.93. Found: 833.63. HPLC-MS (ESI): *m/z* calcd for $C_{26}H_{16}N_2O_4S_5$ $(M+H)^+$: 580.98. Found: 581.43.

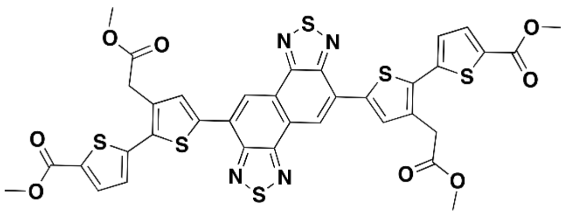

LL-6 methyl ester

Dimethyl 5',5'''-(naphtho[1,2-*c*:5,6-*c'*]bis([1,2,5]thiadiazole)-5,10-diyl)bis(3'-(2-methoxy-2-oxoethyl)-[2,2'-bithiophene]-5-carboxylate)

To a solution of the bromide **2**[4] (0.066 g, 0.177 mmol), 5,10-bis(4,4,5,5-tetramethyl-1,3,2-dioxaborolan-2-yl)naphtho[1,2-*c*:5,6-*c'*]bis([1,2,5]thiadiazole) (0.040 g, 0.081 mmol) and Cs_2CO_3 (0.158 g, 0.484 mmol) in 1,4-dioxane (1.5 mL) was added PEPPSI™-IPr (3.6 mg, 0.005 mmol). After heating the reaction mixture at 75°C for 3h, 1M HCl (aq.) was added until acidic pH. The reaction mixture was diluted with CH_2Cl_2 (400 mL), washed with 2 x 150 mL 1M HCl (aq.); H_2O; 3 x 150 mL sat. $NaHCO_3$ (aq.); H_2O and sat. NaCl (aq.). The organic phase was dried over anhydrous $MgSO_4$, filtered and the solvent was evaporated under reduced pressure to give a dark red/brown/purple colored solid, highly aggregation prone. The solid was washed twice with methanol and centrifuged to collect the pellet **LL-6 methyl ester** (42 mg, 62%). The NMR spectrum can be found at the end of this section.

^{1}H NMR (500 MHz, $CDCl_3$) δ 8.98 (s, 2H), 8.21 (s, 2H), 7.80 (d, *J* = 3.9 Hz, 2H), 7.32 (d, *J* = 3.9 Hz, 2H), 3.93 (s, 6H), 3.92 (s, 4H), 3.81 (s, 6H). HPLC-MS (ESI): *m/z* calcd for $C_{36}H_{24}N_4O_8S_6$ $(M+H)^+$: 833.00. Found: 833.36.

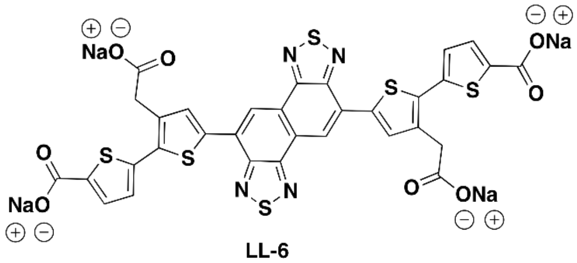

LL-6

Sodium 5',5'''-(naphtho[1,2-*c*:5,6-*c'*]bis([1,2,5]thiadiazole)-5,10-diyl)bis(3'-(carboxylatomethyl)-[2,2'-bithiophene]-5-carboxylate)

NaOH (11 mg, 0.275 mmol) was dissolved in methanol (0.5 mL) and added dropwise to a stirred solution of the tetra methyl ester of LL-6 (19 mg, 0.0228 mmol) in CH_2Cl_2 (5 mL). The reaction was stirred at room temperature for 24 h, diluted with 150 mL H_2O and washed with CH_2Cl_2. The phases were separated and the raspberry red water phase was lyophilized to give the sodium salt of **LL-6** as a purple/brown solid (27 mg, quant.). LL-6 is highly aggregation prone. Therefore, it was not possible to acquire an NMR spectrum of this compound. Instead, we refer to the NMR data for the precursor LL-6 methyl ester. HPLC-MS (ESI): *m/z* calcd for $C_{32}H_{16}N_4O_8S_6$ $(M+H)^+$: 776.94. Found: 777.10.

References

1. A. Åslund, C. J. Sigurdson, T. Klingstedt, S. Grathwohl, T. Bolmont, D. L. Dickstein ¶, E. Glimsdal, S. Prokop, M. Lindgren, P. Konradsson, D. M. Holtzman, P. R. Hof ¶, F. L. Heppner, S. Gandy, M. Jucker, A. Aguzzi, K. Peter, R. Nilsson, *ACS Chem. Biol.* **2009**, *4*, 673–684.
2. T. Klingstedt, A. Åslund, R. A. Simon, L. B. G. Johansson, J. J. Mason, S. Nyström, P. Hammarström, K. P. R. Nilsson, *Organic & Biomolecular Chemistry* **2011**, *9*, 8356.
3. T. Klingstedt, H. Shirani, K. O. A. Åslund, N. J. Cairns, C. J. Sigurdson, M. Goedert, K. P. R. Nilsson, *Chem. Eur. J.* **2013**, *19*, 10179–10192.
4. R. A. Simon, H. Shirani, K. O. A. Åslund, M. Bäck, V. Haroutunian, S. Gandy, K. P. R. Nilsson, *Chemistry - A European Journal* **2014**, *20*, 12537–12543.
5. L. Lantz, H. Shirani, T. Klingstedt, K. P. R. Nilsson, *Chem. Eur. J.* **2020**, *26*, 7425–7432.
6. N. Wahlström, U. Edlund, H. Pavia, G. Toth, A. Jaworski, A. J. Pell, F. X. Choong, H. Shirani, K. P. R. Nilsson, A. Richter-Dahlfors, *Cellulose* **2020**, *27*, 3707–3725.

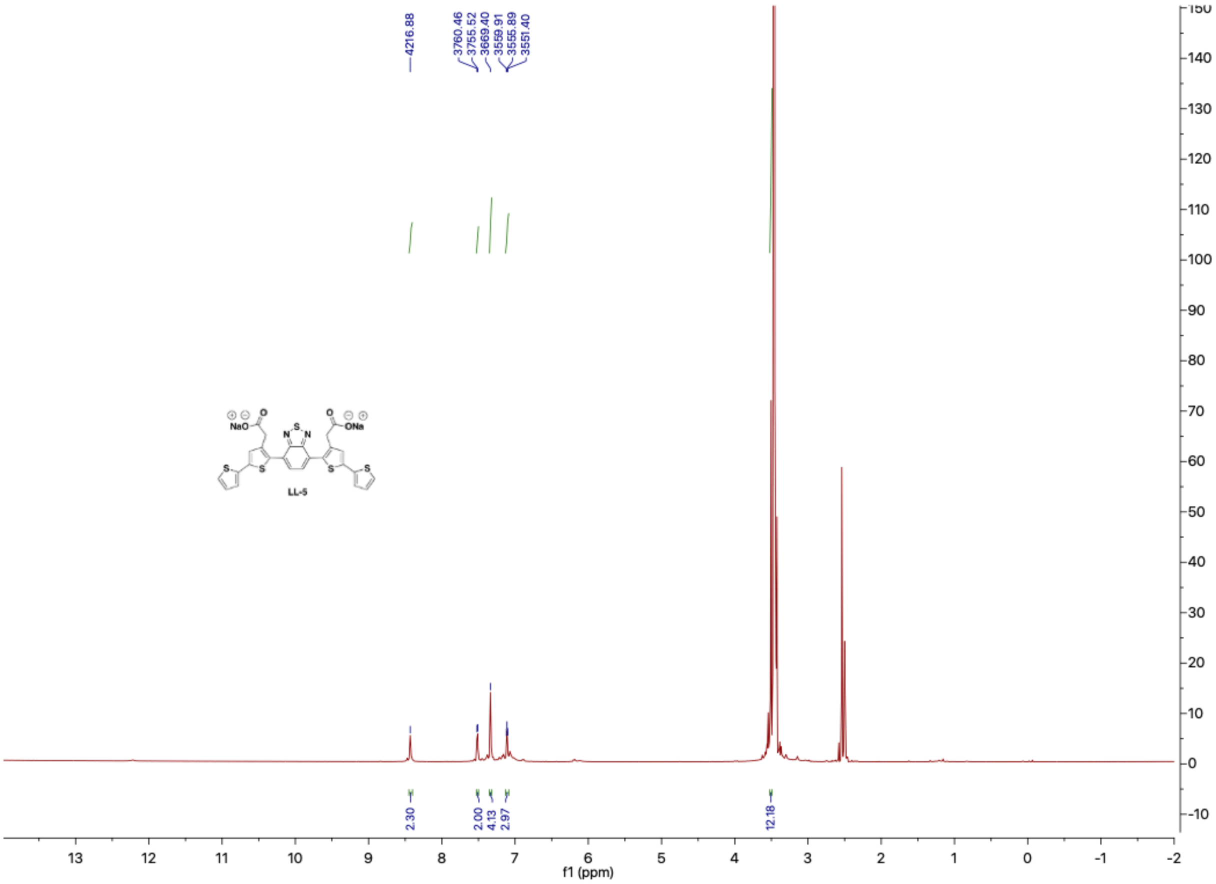

4216.88
3760.46
3755.52
3669.40
3559.91
3555.89
3551.40
NaO
ONa
LL-5
2.30
2.00
4.13
2.97
12.18
150
140
130
120
110
100
90
80
70
60
50
40
30
20
10
0
-10
13
12
11
10
9
8
7
6
5
4
3
2
1
0
-1
-2
f1 (ppm)

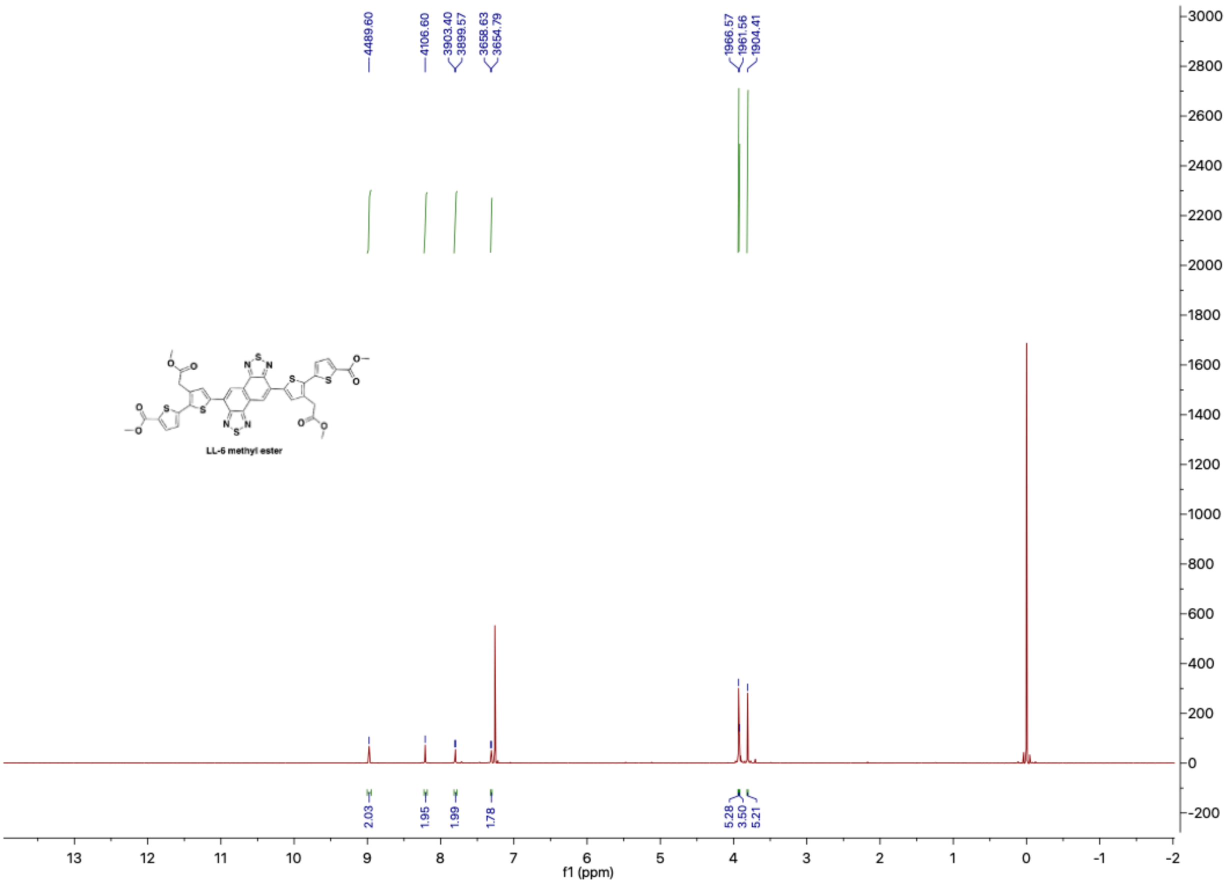
4489.60
4106.60
3903.40
3899.57
3658.63
3654.79
1966.57
1961.56
1904.41
LL-6 methyl ester
2.03
1.95
1.99
1.78
5.28
3.50
5.21
f1 (ppm)
